# Kerosene condenses in the trachea following inhalation

**DOI:** 10.1007/s11419-024-00682-4

**Published:** 2024-03-04

**Authors:** Sella Takei, Hiroshi Kinoshita, Sachiko Kawahara, Mitsuru Kumihashi, Mostofa Jamal, Tadayoshi Yamashita, Etsuko Tanaka, Hiroko Abe, Kunihiko Tsutsui, Shoji Kimura

**Affiliations:** 1 Department of Forensic Medicine, Faculty of Medicine, Kagawa University, 1750-1 Ikenobe, Kita, Miki, Kagawa 761-0793 Japan; 2 Bio Design Inc., Nishi-Ikebukuro, Toshima, Tokyo, 170-0021 Japan; 3 Kagawa Prefectural University of Health Science, 281-1 Hara, Mure, Takamatsu, Kagawa 761-0123 Japan

**Keywords:** Aliphatic hydrocarbon, Tracheal gas phase, Condensation, Kerosene, GC–MS

## Abstract

**Purpose:**

We have investigated the absorption dynamics of petroleum fuel components from the analytical results of autopsy samples.

**Methods:**

Post-mortem samples of the severely burned case, including femoral blood, intratracheal contents (mucus) and intratracheal gas-phase samples were collected, and analysed by gas chromatography-mass spectrometer with head-space solid-phase microextraction.

**Results:**

The composition of flammable substances in the tracheal gas phase differed slightly from that in mucus.

**Conclusion:**

High-boiling point components are retained in the trachea, whereas relatively lower-boiling point components are detected predominantly in the tracheal gas phase and blood.

## Introduction

Petroleum fuel is composed of more than 100 types of hydrocarbons and are used for a variety of purposes, including gasoline, kerosene, light oil, and heavy oil [1]. Petroleum fuels are often detected in fire cases, and a simple screening method is important in forensic practice. Various procedures have been reported as extraction methods for the analysis of petroleum fuels, and solid-phase microextraction (SPME) has been used in recent years [2, 3]. This method can be easily applied for forensic samples without using solvents [2, 3] and is highly useful in forensic practice [4].

Intratracheal gas sampling is useful in cases of fire-related death as a screening test, with results providing valuable information such as whether ignition substances were used or the type of petroleum fuel used [5, 6]. However, there are many unknowns regarding its toxicokinetics. We have investigated the absorption dynamics of petroleum fuel components from the analytical results of autopsy samples, using headspace (HS)-SPME.

## Case history

The severely burned body of a man in his eighties (height, 178 cm; weight, 56 kg) was found in a field with a plastic container holding kerosene nearby. Autopsy findings indicated no evidence of external injury other than broad third- to fourth-degree skin burns with carbonization. The heart weighed 334 g and contained 300 ml of dark-red blood without coagulation. The left and right lungs weighed 522 g and 585 g, respectively, and were congested. A small amount of soot was present in the trachea and bronchi, and airway burns were also observed. Marked congestion was observed in each organ. A drug screening test using an IVeX-screen® panel (Bio Design Inc., Tokyo, Japan), ethanol determination by routine HS-gas chromatography (GC), and routine toxicological examination using liquid chromatography-tandem mass spectrometry were performed [7]. Carboxyhemoglobin saturation level was measured using an AVOX 4000 oximeter (International Technidyne Corporation, Piscataway Township, NJ) [8]. Post-mortem samples were collected for toxicological examinations, including femoral blood, intratracheal contents (mucus) and intratracheal gas-phase samples. Intratracheal gas was collected by direct puncture of the tracheal wall using a syringe with a needle (Terumo, Tokyo, Japan), following the trachea was exposed by frontal neck incision at autopsy [6]. The collected intratracheal gas was immediately prepared in a 20 ml glass vial sealed with a silicone-rubber septum and aluminium cap.

## Equipment and sample preparation for analysis of volatile hydrocarbons

Volatile hydrocarbons were analysed in accordance with a previous report [6]. In brief, a GC-mass spectrometer (QP-2010 Plus; Shimadzu, Kyoto, Japan) fitted with a DB-5MS column (30 m × 0.25 mm I.D., 0.25-µm film thickness; Agilent Technologies, Santa Clara, CA) was used. Each separated compound was identified based on retention time and confirmation ion.

HS-SPME was conducted according to a previous report [6]. The SPME fiber assembly (polydimethylsiloxane, 100 µm thick; Sigma-Aldrich Japan, Tokyo, Japan) was inserted into the HS of the glass vial through the septum. The vial was heated at 60 °C for 15 min in a heating block and the fiber was exposed to the HS of the intratracheal gas sample at room temperature for 15 min. The vial containing the intratracheal gas phase underwent the HS-SPME procedure, as described [6]. Blood or mucus samples (0.5 g) were mixed with 0.5 ml of distilled water, placed in a 20 ml glass vial and sealed with a silicone-rubber septum and aluminium cap. The HS-SPME procedure was applied following these preparations.

## Results

**Fig. 1:**
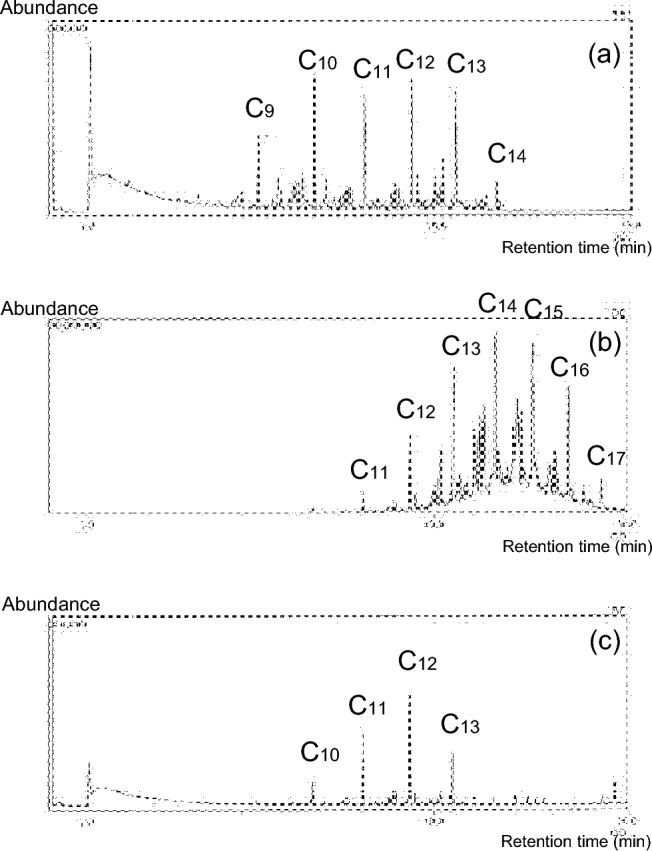
Total ion chromatograms of the intratracheal gas phase (**a**), intratracheal contents (mucus) (**b**) and femoral venous blood (**c**). The intratracheal gas phase sample shows *n-*nonane (C_9_), *n-*decane (C_10_), *n-*undecane (C_11_), *n-*dodecane (C_12_), *n-*tridecane (C_13_) and *n-*tetradecane (C_14_). C_11_–C_14_, *n-*pentadecane (C_15_), *n-*hexadecane (C_16_) and *n-*heptadecane (C_17_) are detected in the mucus sample. C_10_–C_13_ are identified in the femoral venous blood sample

Figure 1 shows total ion chromatograms for intratracheal gas phase, mucus, and femoral venous blood samples from the deceased. Each peak in the intratracheal gas sample was identified as a saturated aliphatic hydrocarbon, including *n-*nonane (C_9_), *n-*decane (C_10_), *n-*undecane (C_11_), *n-*dodecane (C_12_), *n-*tridecane (C_13_) and *n-*tetradecane (C_14_). C_10_–C_13_ were also identified in the blood sample. These results indicated that the deceased had inhaled kerosene prior to death [6, 9–11]. Aliphatic hydrocarbons such as *n-*pentadecane (C_15_), *n-*hexadecane (C_16_) and *n-*heptadecane (C_17_) were detected in the mucus in addition to C_11_–C_14_.

## Discussion

The intensity pattern of aliphatic hydrocarbons in the mucus differed from that in the intratracheal gas phase, but was similar to that of kerosene itself [12]. Relatively high-boiling point (BP) components (C_15_–C_17_) were observed in mucus, while the main components in the gas phase shifted to low-BP components, suggesting condensation within the trachea, as pointed out by Yoshida et al. [12]. For this reason, low-BP compounds were predominantly detected in the blood. It has been reported that hydrocarbons with C_14_ or high BP components were hardly inhaled [13]. This result also supports our findings. The detailed absorption dynamics following exposure to kerosene have not been elucidated, and the present results seem to clarify some points. The gas phase is easily collected and results are easy to obtain, so the ability to distinguish between gasoline and kerosene [6] makes this approach highly useful as a screening test.

The IVeX-screen® panel yielded negative results and no ethanol or other substances were detected. Carboxyhemoglobin saturation was 10.3% in the left heart blood sample, 8.9% in the right heart blood sample and 8.5% in the femoral venous blood sample. Based on the autopsy findings, police investigation and toxicological examination, we concluded that the cause of death was burns following the use of kerosene as an ignition accelerator.
